# History and Perspective of Immunotherapy for Pythiosis

**DOI:** 10.3390/vaccines9101080

**Published:** 2021-09-26

**Authors:** Hanna Yolanda, Theerapong Krajaejun

**Affiliations:** 1 Section for Translational Medicine, Faculty of Medicine, Ramathibodi Hospital, Mahidol University, Bangkok 10400, Thailand; hanna.yoa@student.mahidol.ac.th; 2 Department of Parasitology, School of Medicine and Health Sciences, Atma Jaya Catholic University of Indonesia, Jakarta 14440, Indonesia; 3 Department of Pathology, Faculty of Medicine, Ramathibodi Hospital, Mahidol University, Bangkok 10400, Thailand

**Keywords:** pythiosis, *Pythium insidiosum*, antigen, treatment, immunotherapy

## Abstract

The fungus-like microorganism *Pythium insidiosum* causes pythiosis, a life-threatening infectious disease increasingly reported worldwide. Antimicrobial drugs are ineffective. Radical surgery is an essential treatment. Pythiosis can resume post-surgically. Immunotherapy using *P. insidiosum* antigens (PIA) has emerged as an alternative treatment. This review aims at providing up-to-date information of the immunotherapeutic PIA, with the focus on its history, preparation, clinical application, outcome, mechanism, and recent advances, in order to promote the proper use and future development of this treatment modality. *P. insidiosum* crude extract is the primary source of immunotherapeutic antigens. Based on 967 documented human and animal (mainly horses) pythiosis cases, PIA immunotherapy reduced disease morbidity and mortality. Concerning clinical outcomes, 19.4% of PIA-immunized human patients succumbed to vascular pythiosis instead of 41.0% in unimmunized cases. PIA immunotherapy may not provide an advantage in a local *P. insidiosum* infection of the eye. Both PIA-immunized and unimmunized horses with pythiosis showed a similar survival rate of ~70%; however, demands for surgical intervention were much lesser in the immunized cases (22.8% vs. 75.2%). The proposed PIA action involves switching the non-protective T-helper-2 to protective T-helper-1 mediated immunity. By exploring the available *P. insidiosum* genome data, synthetic peptides, recombinant proteins, and nucleic acids are potential sources of the immunotherapeutic antigens worth investigating. The PIA therapeutic property needs improvement for a better prognosis of pythiosis patients.

## 1. Introduction

Pythiosis is a life-threatening infectious disease [1,2,3,4,5,6,7] that has been increasingly reported in humans [8,9,10,11,12,13], horses [14,15,16,17,18,19], dogs [20,21,22,23], cats [24,25,26,27], camels [28,29], and some other animals [30,31,32,33,34] living in tropical and subtropical areas worldwide. The causative agent is the oomycete microorganism *Pythium insidiosum*, which inhabits water and moist soil [35,36,37,38,39,40]. Biflagellate zoospore is an infective stage of *P. insidiosum* that shows strong tropism to animal hairs and tissues [41,42]. Direct exposure to the pathogen habitat (i.e., stagnant water, rice field, pond) could increase the risk of the infection [43,44,45,46,47]. Patients with pythiosis usually manifest with clinical features associated with an infection of the skin [11,23,48,49], artery [13,50,51], eye [47,52,53,54], gastrointestinal tract [28,55,56,57], or another internal organ [5,12,58,59,60]. Diagnostic methods have been established for pythiosis [61], such as culture identification [62,63,64], histopathological examination [65,66,67,68], serological assays [69,70,71,72,73,74,75], molecular techniques [76,77,78,79,80,81,82], and proteomic analysis [83,84].

Prompt and effective treatment improves the patient’s prognosis [56,85,86,87,88,89,90,91,92,93,94]. The use of conventional antifungal drugs provides limited efficacy in treating pythiosis because *P. insidiosum* lacks the drug-target sterol biosynthesis enzymes [95]. A few antibacterial drugs demonstrate a favorable outcome in some pythiosis patients [53,90,96,97]. However, several in vitro and in vivo studies show that these antimicrobial agents exhibit diverse inhibitory effects among different strains of *P. insidiosum* [98,99,100,101,102,103,104]. Therefore, radical surgery to remove all infected tissues is the primary treatment option for pythiosis [44,50,60,105,106]. However, the surgical procedure could be challenging and complex in some pythiosis patients with disseminated [12,43,58], cerebral [2,107,108], or complicated vascular infection [90,109,110]. Besides, pythiosis can recur post-surgically. New management tools to treat the infections caused by *P. insidiosum* are needed.

Alternative treatment options for pythiosis, i.e., biogenic silver nanoparticles (Ag-NP), photodynamic therapy (PDT), and ozone have been explored [111,112,113,114,115,116,117,118]. Valente et al. show that Ag-NP inhibits *P. insidiosum* in the rabbit model of pythiosis [112]. Pires et al. reveal that the photosensitizer-based PDT reduces the growth of *P. insidiosum* [114,115]. Ozone gas and ozonated sunflower oil inhibit the growth of *P. insidiosum* and cure some horses with pythiosis [116,117,118]. More studies are required to demonstrate the in vivo effectiveness and safety of Ag-NP, PDT, and ozone application against *P. insidiosum*. The *P. insidiosum* antigen (PIA) immunotherapy had been used for decades to cure pythiosis in different species [119,120,121,122,123]. The immunomodulating PIA is usually administered in combination with other therapeutic approaches (i.e., surgery and antimicrobial drugs) [13,106,109,121,124,125,126,127] and shows a favorable response in many pythiosis patients [13,119,128,129,130,131,132]. Nevertheless, the true therapeutic efficacy of PIA alone is unknown.

This review aims at providing up-to-date information of the immunotherapeutic PIA, with a focus on its history, preparation, clinical application, outcome, mechanism, and recent advances, in order to promote the proper use and future development of this treatment modality. The keywords “pythiosis” and “*Pythium insidiosum*” were used to search the literature (i.e., PubMed, SciELO, and Google Scholar) up until June 2021 for compiling relevant PIA information. Furthermore, the information relating to PIA was obtained from the Google patent database browser (https://patents.google.com/; accessed on 29 May 2021) and the United States Patent and Trademark Office website (https://www.uspto.gov/; accessed on 29 May 2021). In the last part, we prospected the future development for improving the effectiveness of the PIA immunotherapy.

## 2. History of the Immunotherapy Using *P. insidiosum* Antigens

The use of PIA in pythiosis immunotherapy was first reported in Australia in 1981 by Miller et al., who applied this treatment modality in affected animals since 1977 [119]. The PIA formulation containing cytoplasmic antigens (prepared by sonication of *P. insidiosum* hyphae) showed a cure rate of 53% (21 out of 40 affected horses) [119]. However, a calf with pythiosis was treated with the same PIA but showed no response [133]. Limitations of this antigen formulation include short shelf life (up to 8 weeks), unresponsiveness in cases with a long history of the *P. insidiosum* infection (i.e., 3.5 and 7 months), and skin reaction or sterile abscess at the injection site in 30–70% of the horses [119,126].

In 1986, Mendoza et al. reported a different PIA formulation for treating five horses with cutaneous pythiosis in Costa Rica [120]. Their formulation contains culture filtrate antigens (CFA; representing exoantigens or extracellular proteins) prepared by ether precipitation of *P. insidiosum* hyphae-removed culture broth [120]. It cured three out of five horses with pythiosis (60%), exhibited a relatively long shelf life (~8 months), and demonstrated minimal skin reaction with no sterile abscess at the injection site. However, it did not show any favorable response in the horses with chronic *P. insidiosum* infection [120].

In 1992, Mendoza et al. described two new PIA formulations for immunotherapy of equine pythiosis, so-called: (i) soluble concentrated antigen vaccine (SCAV) and (ii) cell mass vaccine (CMV) [123]. SCAV contains CFA from acetone precipitation of *P. insidiosum* hyphae-removed culture broth, whereas CMV contains antigens from homogenized *P. insidiosum* hyphae [123]. Compared with CMV, SCAV showed a higher cure rate [29 of 41 affected horses (71%) vs. 18 of 30 affected horses (60%)] and longer shelf life (18 months vs. 2–3 weeks). In addition, SCAV exhibited less skin reaction at the injection site than did CMV. Besides, sterile abscesses were noted in about half of 30 CMV-immunized horses [123]. Although SCAV improved the treatment outcome, it did not cure the horses with chronic pythiosis (a prolonged infection of more than 2 months) [123].

Western blot analysis of the cytoplasmic proteins of *P. insidiosum*, so-called soluble antigens from broken hyphae (SABH; prepared by sonication of hyphae), showed 3 major immunodominant antigens at the molecular weights of 28, 30, and 32 kDa [134,135]. Mendoza et al. added these antigens into SCAV (containing exoantigens) [135,136]. All antigens were prepared from the *P. insidiosum* isolate ATCC 74446 (also known as the isolate ATCC 58643 or CBS 574.85) [106,136]. The resulting PIA formulation showed enhanced efficacy, as it cured 13 out of 18 horses with cutaneous pythiosis (72%), including 5 horses with a chronic skin lesion of at least 4 months [121], and a human patient with vascular pythiosis [107]. From 6 dogs with cutaneous and intestinal pythiosis, it cured 2 cases (33%) while exhibited no favorable response in 4 cases (67%; including 4 dogs with a chronic lesion longer than 2 months) [121]. It cannot cure cutaneous pythiosis in a cat [137]. This PIA formulation, during its development, had been sequentially patented in 1999, 2001, 2004, and 2010 under the U.S. patent numbers 5948413, 6287573B1, 6833136B2, and 7846458B1, respectively [136,137,138], and was made commercially available in the United States by Pan American Veterinary Laboratories, Texas, USA [125,139,140,141].

In 2003, Santurio et al. developed and compared the efficacy of three PIA formulations, containing (i) vortexed (or macerated) [142,143], (ii) sonicated, or (iii) mixed (vortexed and sonicated) hyphal antigens of *P. insidiosum*, in the rabbit model of pythiosis [122]. The method used to prepare the sonicated antigens of Santurio et al. is similar to Miller’s, except the former lyophilized the extracted proteins to prolong the shelf life at room temperature [119,122]. The vortexed hyphal antigens were obtained by vortexing *P. insidiosum* hyphae in the presence of sulfuric ether [122]. After lyophilization, the vortex-derived PIA was stable for more than one year [122]. Using the rabbit model of pythiosis in a case-control study (four groups of five rabbits), the PIA formulation containing the vortexed hyphal antigens outperformed the placebo, and the PIA formulation containing sonicated or mixed hyphal antigens [122]. The rabbit group that received the vortexed antigens showed a 72% reduction in lesion size (compared with the lesion size before treatment), and 2 rabbits of this group were considered clinically and histologically cured. In contrast, the other groups of rabbits treated with placebo and other antigens had an enlarged lesion (up to 212% increase than the lesion size before treatment). Besides, the use of vortexed antigens was evaluated in 35 horses with pythiosis [144]. It showed no disease-prevention property while demonstrating a curative property in 74% of the affected horses with a recent or chronic lesion [144]. The PIA formulation containing vortexed antigens is commercially available by the Laboratório de Pesquisas Micológicas-Universidade Federal de Santa Maria (LAPEMI-UFSM), Brazil.

Later in 2011, Mendoza et al. modified their PIA formulation by using the *P. insidiosum* isolate MTPI-04 (type isolate: ATCC PTA-12166) as the antigen source and introducing a cryogenic method to disrupt the hyphal elements (i.e., grinding the hyphae in a mortar in the presence of liquid nitrogen) [145,146]. Western blot analysis showed that this PIA preparation contains the additional 124 kDa protein and a greater quantity of the 28 kDa protein [145,146]. In 2015, Permpalung et al. compared the efficacies of 2 PIA formulations prepared from different *P. insidiosum* isolates (MTPI-04 vs. ATCC 58643) in human patients with pythiosis [106]. Both PIA formulations, comprising CFA (extracellular antigens) and SABH (intracellular antigens), showed no significant differences in clinical outcomes for patients with vascular or ocular infection.

## 3. Preparation of *P. insidiosum* Antigens for Pythiosis Immunotherapy

Although the PIA formulations for pythiosis immunotherapy vary, all contain either extracellular proteins (so-called CFA, exoantigens, or secretory antigens) [43,61,107,121,131,135,136,137,138] and/or intracellular proteins (so-called cytoplasmic antigens or endoplasmic antigens) [43,61,121,136,137,138], extracted from a selected isolate of *P. insidiosum*, using a protocol of choice. In brief, the organism is cultured in a liquid medium (i.e., Sabouraud dextrose or nutrient broth), with or without shaking (100–150 rpm), at 37 °C for 5–10 days [119,120,122,123,136]. Growing *P. insidiosum* hyphae are harvested from the liquid culture by discarding the fluid [119] or filtrating through a membrane [123] to prepare cytoplasmic antigens. The remaining cell-free culture broth is collected for the preparation of exoantigens. The harvested *P. insidiosum* hyphae are ruptured to release the cytoplasmic antigens by using one of the following tools: cell homogenizer [123], sonicator [119,122,136], or vortex shaker [122]. The hyphal cell lysate (resuspended in water, saline, or phosphate-buffered saline pH 7.2) is centrifuged to collect the supernatant containing soluble cytoplasmic antigens [119,122,136,138]. As a part of exoantigen preparation, an appropriate amount of ether (i.e., an ether-to-broth ratio of 1:1) or acetone (i.e., an acetone-to-broth ratio of 1:1 or 2:1) is added to the cell-free culture broth to precipitate the extracellular proteins of *P. insidiosum* [120,123,136,138]. The precipitated exoantigens are collected by centrifugation.

Some investigators combined different antigens to formulate a new PIA. For example, Santurio et al. mixed the cytoplasmic antigens prepared by different methods (i.e., vortex and sonication) [122], whereas Mendoza et al. merged the cytoplasmic antigens and exoantigens extracted from the same isolate [136,138]. Inactivation of *P. insidiosum*, before or after the protein extraction, can be achieved using 0.02% thiomersal or 0.5% phenol [119,122,123,136]. Microbial contamination is checked by spreading 100 µL of the crude extracted proteins onto Sabouraud dextrose and blood agar plates before incubation at 37 °C for one week [119,123,136]. The obtained PIA can be kept at 4 °C for short-term storage (i.e., several weeks to months) or at freezing temperature (i.e., −21 or −80 °C) for a longer period [106,119,120,123,127,131,135,136]. Lyophilization can preserve the extracted antigens at room temperature for at least one year [122].

## 4. Clinical Application of Immunotherapy in Human Pythiosis

Primary clinical forms of human pythiosis include vascular pythiosis (an infection of a medium-to-large artery) and ocular pythiosis (an eye infection). A few patients come with cutaneous pythiosis (an infection of the skin) [43,147,148] or disseminated pythiosis (an infection of multiple organs) [12,43,60]. Surgical intervention is the primary treatment of pythiosis [13,61,106,109,127,132,149,150]. The therapeutic goal is to remove all infected tissues to achieve an organism-free surgical margin [13,90,106,109,151,152]. The first use of PIA immunotherapy in a human patient with pythiosis was reported in 1998 [107]. After being treated with PIA immunotherapy, this patient has recovered from the *P. insidiosum* infection of external and internal carotid arteries, where radical surgery is impossible. After excluding potentially-overlapping or clinical data-lacking cases (i.e., no treatment outcome), the literature search identified 108 patients with vascular pythiosis (Table 1), 35 patients with ocular pythiosis (Table 2), and 2 patients with a periorbital infection that received the PIA immunotherapy as an adjunctive treatment, together with antimicrobial drugs or surgery [2,7,13,43,90,106,107,108,109,131,132,149,150,151,152,153,154,155,156,157,158,159,160]. The PIA formulation used in these patients contained both extracellular and intracellular proteins of *P. insidiosum* [136,137,138]. The final clinical outcomes of these 145 PIA-immunized patients were “cured” in 84.1% of cases (with or without losing an affected organ; *n* = 122) and “dead” in 15.9% of cases (*n* = 23).

Among 39 vascular pythiosis patients who did not receive PIA immunotherapy, the majority (59.0%; *n* = 23) survived the disease, while the remaining (41.0%; *n* = 16) died from an advanced infection (Table 1). On the other hand, from 108 vascular pythiosis patients who received the PIA immunotherapy, most cases (80.6%; *n* = 87) survived, whereas the rest (19.4%; *n* = 21) died (Table 1). Regardless of clinical settings (i.e., infection site and disease severity), PIA immunotherapy seemingly improved the survival rate by ~20% in patients with vascular infection. Clinical details (i.e., ages, genders, underlying conditions, extents of an affected artery, and clinical outcomes) were available from 105 vascular pythiosis patients with (67 out of 108 cases; 62.0%) and without (38 out of 39 cases; 97.4%) PIA immunotherapy (Table 3). Both groups showed comparable ages (years: 40.9 vs. 41.4), underlying illnesses (hematological diseases: 91.0% vs. 100.0%), infection sites (legs: 92.5% vs. 100.0%), and morbidity (amputations: 61.2% vs. 57.9%). The overall mortality rate of the patients with PIA immunotherapy (29.9%) was lower than those without (42.1%) (Table 3).

The *P. insidiosum* infection usually progresses along an affected artery from the distal to the proximal part of the leg. Pythiosis involving the aorta and iliac artery possessed a remarkably high mortality rate of 88.2% in the patients without PIA immunotherapy and a relatively lower rate of 61.9% in the patients with such treatment (Table 3). Regardless of PIA immunotherapy, the mortality rate appeared to be lower (up to 15.2%) in the pythiosis patients with an infection of a lower-level artery, such as femoral, popliteal, tibial, peroneal, and dorsalis pedis arteries. Six PIA-immunized patients survived vascular pythiosis without leg amputations [13,106,107,131] (Table 3).

Concerning the disease duration, up to 2 months before treatment, the PIA-immunized and PIA-unimmunized vascular pythiosis patients showed similar mortality rates (28.6% vs. 25.0%; Table 3). However, if the disease duration was longer than 2 months (described as chronic infection by Mendoza et al. [121]), the PIA-immunized patients had ~17% lower mortality rate than the PIA-unimmunized cases (33.3% vs. 50.0%; Table 3). Thus, a longer disease duration could lead to a higher mortality rate, and PIA immunotherapy could notably reduce disease mortality in patients with a chronic *P. insidiosum* infection.

Because the PIA-immunized patients usually obtained a combination treatment (including surgery and antimicrobial agents), the sole immunotherapy efficacy against *P. insidiosum* cannot be evaluated directly. The PIA-immunized patients with a marked inflammatory reaction (i.e., local swelling, pruritus, and erythema at the injection site and regional lymphadenopathy) showed a more favorable treatment outcome than patients with minimal or no such reaction [106,107,131,157]. Several serum markers, such as β-D-glucan (BDG), anti-*P. insidiosum* antibodies, erythrocyte sedimentation rate (ESR), and C-reactive protein (CRP), have been used to monitor the clinical course of some vascular pythiosis patients [90,151,164]. A declining level of BDG [90,151], ESR [164], or CRP [164] links with a recovery condition in the course of pythiosis treatment. Lower serum BDG and higher anti-*P. insidiosum* antibodies are associated with a better prognosis [151].

Similar to vascular pythiosis, surgical intervention is also the primary option for treating ocular pythiosis [132,150,167,177,180,181,184,185]. Topical or systemic antimicrobial agents (including linezolid, azithromycin, itraconazole, terbinafine, amphotericin B, natamycin, moxifloxacin, and minocycline) were administered in the ocular cases [97,106,174,180,181,182,186,187]. In addition, many ocular pythiosis patients underwent penetrating keratoplasty to save the affected eye [53,54,91,165,166,168,170,173,176,183]. Post-keratoplasty recurrent infection resulted in eye removal by evisceration or enucleation [66,91,150,176,184]. In addition to the surgical and medical treatments, PIA was administered to modulate the immune response in 35 out of 168 ocular pythiosis patients (20.8%; one of which had bilateral ocular infections) [1,106,127,132,150,180] and 2 out of 5 periorbital pythiosis patients (40.0%) [43,160] (Table 2). The affected eye was removed in 17 out of 35 PIA-immunized (48.6%) and 47 out of 133 PIA-unimmunized (35.3%) patients (Table 2). Regarding the patients with periorbital pythiosis (*n* = 5), one of the two cases with, and all three cases without, PIA immunotherapy survived the disease. One each of the ocular and periorbital pythiosis patients, who obtained PIA immunotherapy, died due to an invasive infection [1,43]. Based on these reports, the PIA immunotherapy may not provide an advantage in a local *P. insidiosum* infection of the eye.

Administration of the PIA immunotherapy for pythiosis patients (i.e., antigen concentration, injection site, number of doses, and duration between shots) can differ based on the clinician’s judgment and PIA availability. The immunotherapeutic PIA is generally prepared to the final concentration of 2 mg/mL [90,106,107,127,131,132,151,180]. Either 0.1–0.2 mL [43,106,107,131,132] or 1.0 mL [90,106,109,151,180] of PIA per dose is injected subcutaneously at least 2 times: initial and subsequent time points (i.e., 0.5, 1, 1.5, 3, 6, and 12 months) [13,43,61,106,107,109,127,131,132,149,151,180]. In some cases, the first PIA dose was provided intradermally [106,131]. Several patients with an aggressive *P. insidiosum* infection obtained a PIA injection once a week up to 7 weeks [7,106,156]. Sermsathanasawadi et al. describe the immunotherapy outcome of some patients with vascular pythiosis and recommend to provide an affected patient 3 PIA injections at days 0, 7, and 21 [109].

## 5. Immunotherapy Using *P. insidiosum* Antigen in Animals with Pythiosis

Initially, PIA was formulated and modified to increase its clinical efficacy in the immunotherapy of horses with pythiosis [119,120,121,122,123,188,189,190]. Besides, PIA has been used to treat the disease in other animals, such as dogs [121,125,129,140,141,191,192], camels [29,124], calves [130,133], sheep [193], cats [25,27,137,194], and a donkey [128] (Table 4). Several reports show that PIA immunotherapy, used as the primary treatment, can cure pythiosis in animals [128,130,192,193,195]. However, many affected animals required such immunotherapy combined with surgical and antimicrobial treatments [124,126,128,129,140]. In general, clinical outcomes can vary among the animals with pythiosis who received PIA immunotherapy, either alone or in combination with other treatments. In most cases, PIA (1.0–2.5 mL/dose) was injected subcutaneously, every 7–15 days, usually until the lesion resolved [25,118,124,128,129,130,140,193,195,196,197]. Some infected animals received the first dose of PIA intradermally and the subsequent doses subcutaneously [121,192]. The immunotherapeutic PIA can be injected at the pectoral muscle [119,123,126,198,199], neck [120,121,123,144,197], or shoulder [192] of an animal. A mild-to-extended inflammatory reaction can be noticed at the injection site [29,192,197,198,199].

By excluding overlapping and clinical data-lacking cases, a total of 270 animals with pythiosis (including 239 horses, 15 dogs, 6 sheep, 4 cats, 3 camels, 2 calves, and a donkey), who received the PIA immunotherapy, were identified in the literature (Table 4). The horse is the most affected species for pythiosis. All 239 horses immunized with PIA had a *P. insidiosum* infection of the skin (several cases had a co-infection of bone, lung, or liver) (Table 4 and Table 5), 167 of which (69.9%) were clinically cured with (38 from 167 cured cases; 22.8%) or without (129 from 167 cured cases; 77.2%) a surgical intervention. For comparison, of 170 PIA-unimmunized horses with pythiosis (Table 5), 125 (73.5%) were cured with (94 from 125 cured cases; 75.2%) or without (31 from 125 cured cases; 24.8%) surgery. Regarding dogs with cutaneous or gastrointestinal pythiosis, the clinical cure was observed in 6 out of 15 PIA-immunized cases (40.0%) and 17 out of 75 PIA-unimmunized cases (22.7%) (Table 4 and Table 5). Relatively low clinical efficacy of the PIA immunotherapy was observed in other animals (Table 4). For example, only 1 out of 6 sheep (16.7%) [193], 1 out of 3 camels (33.3%) [29,124], and 1 out of 2 calves (50.0%) [130,133] were cured of pythiosis. Besides, the PIA immunotherapy provided a negative response in 4 cats and a donkey with cutaneous pythiosis (Table 4). Notably, the clinical cure was observed in 127 out of 128 infected cows without PIA immunotherapy (99.2%) (Table 5). It should be noted that a recurrent *P. insidiosum* infection was observed in some PIA-immunized horses previously cured of pythiosis [128,206], suggesting that the natural infection and PIA may not induce adequate or sustainable protective immunity [144].

Regardless of the clinical condition and management, the horses receiving PIA immunotherapy showed a slightly lower cure rate than those not receiving such treatment (69.9% vs. 73.5%) (Table 5). In contrast, the PIA-immunized dogs exhibited a markedly higher cure rate than the PIA-unimmunized cases (40.0% vs. 22.7%) (Table 5). Some animal species (i.e., sheep, camel, cat, and donkey), those that appeared to be less affected by *P. insidiosum*, had a relatively low favorable response (from none to 33.3%) to PIA immunotherapy. From 130 recruited cows with pythiosis, only 2 were treated with PIA, resulting in 1 cured case (Table 5). Notably, most infected cows (127 from 128 cases; 99.2%) were spontaneously recovered from pythiosis, suggesting a potent host immunity against *P. insidiosum* generated in this particular animal species. A question has arisen concerning the immunotherapy efficacy since the overall cure rate for the infected animals who received PIA (as a part of their treatments) was lower than those who did not receive the antigen: 65.2% (176 in 270 cases) vs. 71.9% (271 in 377 cases) (Table 5). However, it is uncertain whether the clinical conditions of the affected animals with PIA immunotherapy were more severe than those without it, resulting in a relatively worse prognosis. Nevertheless, among the horses who were cured of pythiosis, it appears that the surgical interventions took place in 75.2% of the PIA-unimmunized cases. The rate of such interventions dropped to 22.8% in the PIA-immunized horses, suggesting PIA immunotherapy could reduce disease morbidity. As with human pythiosis, unless there is a case-control study, the favorable efficacy of PIA immunotherapy in animals cannot be directly assessed due to the different clinical settings among the cases, such as underlying condition, disease onset, severity, lesion size, pathologic location, and choices of treatment.

## 6. Proposed Mechanism of *P. insidiosum* Antigen-Based Immunotherapy

During the *P. insidiosum* infection, an antigen-presenting cell (APC), such as a dendritic cell, could process and present a pathogen antigenic epitope (via major histocompatibility complex or MHC) to a naïve T cell (through T-cell receptor or TCR). Such APC-T cell interaction induces differentiation and clonal proliferation of a naïve CD4+ T (Th0) cell to T helper-2 (Th2) cells [142,242,243,244]. An elevated level of IL-4 is responsible for the differentiation and proliferation of Th2 cells, which, in turn, secretes IL-5 for activating eosinophils [131,142,245,246]. This process leads to the non-protective Th2-mediated immunity, where eosinophils are predominantly recruited, together with other cell types, such as mast cells, neutrophils, giant cells, and plasma cells, into the infection area [18,28,66,160,199,217,247]. The eosinophils surround the *P. insidiosum* hyphae inside necrotic tissues, producing the histological phenomenon called “Splendore-Hoeppli” [49,105,248,249,250]. *P. insidiosum* might employ the accumulated eosinophilic materials as a protective shield against host immunity [122].

**Figure 1:**
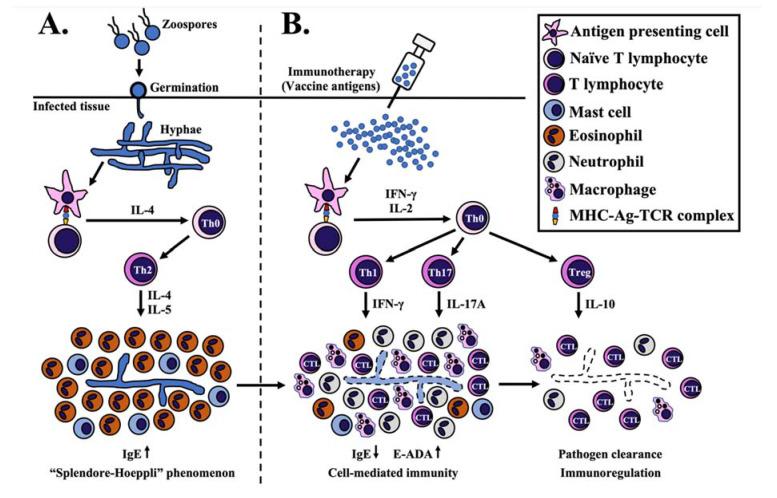
Proposed mechanism of *P. insidiosum* antigen (PIA) immunotherapy. (**A**) *P. insidiosum*’s zoospores (asexual stage) attach and germinate as hyphae into the host tissue during natural infection. Antigen-presenting cells (APC) process and present the *P. insidiosum* antigens to naïve T lymphocytes via major histocompatibility complex-antigen-T cell receptor complex (MHC-Ag-TCR). This interaction elevates some cytokines (mainly IL-4) to differentiate and clonally proliferate a naïve CD4+ T (Th0) to T helper-2 (Th2) cell, which, in turn, produces IL-4 and IL-5 to attract and activate eosinophils and mast cells. The eosinophils surround the *P. insidiosum*, creating the histological phenomenon called “Splendore-Hoeppli”. (**B**) Processed antigens (prepared from the crude extract of *P.* *insidiosum*) lead to forming the MHC-Ag-TCR complex that induces the release of some cytokines, mainly IFN-ϒ and IL-2. IFN-ϒ promotes differentiation and clonal proliferation of a Th0 to T helper-1 (Th1) cell. Th1 cell-produced IFN-ϒ attracts macrophages and cytotoxic T lymphocytes (CTL) to the infection site for eliminating the pathogen. The MHC-Ag-TCR complex also facilitates the differentiation of a Th0 to T helper 17 (Th17) cell to produce IL-17A and accumulate more macrophages and neutrophils at the infection area. On the other hand, IL-2 promotes the differentiation of a Th0 to regulatory T (Treg) cell to regulate or suppress an excessive immune response through the release of IL-10. See the text for the details and the references to the proposed mechanism of PIA immunotherapy.

The PIA immunotherapy shows a favorable response in some humans and animals with pythiosis [46,106,129,130,131,180]. The mechanism of PIA action in pythiosis treatment is not clearly understood. However, recovery of the PIA-immunized humans and animals from pythiosis is associated with switching Th2 to T helper-1 (Th1) mediated immunity [131,142,244,251,252]. An APC should process an immunomodulating antigen of *P. insidiosum*, such as (1,3)(1,6)-β-glucan, which is a main cell wall component [251,253,254], before priming a resulting antigenic epitope to a naïve T cell (via MHC-TCR complex) [242,243]. This cellular interaction might trigger the release of IFN-γ and IL-2 for enhancing the differentiation of Th0 to Th1 cell and Th0 to regulatory T (Treg) cell, respectively [242,246,251,252,255,256]. The Th1 cell produces IFN-γ to activate the macrophages and cytotoxic T lymphocytes [131,142,242,244,246,251,253,256]. The PIA raises the T helper-17 (Th17) cells, which secrete IL-17A to recruit neutrophils and macrophages into the infected tissue [242,243,246,251,252,253]. The PIA could also promote the release of the IL-10 cytokine, which relates to the anti-inflammatory and immunoregulation activity of Treg cells [242,246,251,252,253,256]. In the rabbit model of pythiosis, the PIA elevates ecto-adenosine deaminase (E-ADA), which could stimulate the purinergic signaling system and promote the Th1-mediated immunity [257,258,259]. After PIA immunotherapy, histological findings at the infection site include the recruitment of lymphocytes, the gradual absence of eosinophils, and the reduced tissue burden of *P. insidiosum* hyphae [121,122,250,257]. The immunoglobulin E (IgE), which is increased during the *P. insidiosum* infection, is then decreased following the immunotherapy [121,131,244]. Taken together, the proposed *P. insidiosum* clearance mechanism of PIA immunotherapy is summarized in Figure 1 [131,142,242,244,251,253,259]. The in-depth mechanism of the immunomodulating PIA action in pythiosis treatment needs further investigation.

The PIA immunotherapy can cure many affected human and animal patients with pythiosis [13,106,149]. However, several pieces of evidence indicate that the host immunity induced by PIA could not prevent the *P. insidiosum* infection [46,121,122,144]. For example, Santurio et al. demonstrate no difference in the incidences of pythiosis between PIA-immunized and PIA-unimmunized horses [144]. Besides, several reports show that pythiosis can recur in recovered patients with or without PIA immunotherapy [45,123,125,188,206]. These findings suggest that the host immunity, induced by either immunotherapy or natural infection, may be inadequate in preventing another episode of *P. insidiosum* infection. Nevertheless, *P. insidiosum* reinfection could induce a stronger host immune response (i.e., a higher level of IgG antibodies) than the previous infection [206]. In patients with a persistent infection, the immunotherapy-induced antibody level could be affected by several factors, including host immune status, underlying diseases, and severity of the infection [2,106,201,204]. Although a high level of the anti-*P. insidiosum* antibodies generated by immunotherapy or natural infection is associated with the ability to eliminate the pathogen, the antibodies can be gradually decreased over time to a low or undetectable level [107,131,134,206], explaining the limited protective immunity against reinfection.

## 7. Future Perspective

Pythiosis has high mortality and morbidity. For decades several immunotherapeutic antigen formulations, prepared from crude extracts of *P. insidiosum*, have been used in the management of human and animal pythiosis [1,25,29,106,107,109,119,129,130,131,132,149,180,188,189,190,193,239,260]. However, a prophylactic approach to prevent the infection has yet to be developed. Clinical pieces of evidence show that the PIA immunotherapy can cure some, but not all, humans and animals with pythiosis [13,131,149,151]. The PIA immunotherapy, mostly in conjunction with surgery and antimicrobial drugs, demonstrates different clinical outcomes from study to study, likely depending on the severity of the *P. insidiosum* infection, host immune status, PIA formulations, the strain used for antigen preparation, and affected host species (i.e., human, horse, dog, cat, and cattle). Understanding how the PIA modulates the host immune responses to eliminate *P. insidiosum* could lead to developing more effective immunotherapy and, therefore, improving pythiosis patients’ clinical outcomes.

Information on the efficacy of PIA immunotherapy against pythiosis has been obtained, based mainly on clinical observation of the affected patients. However, no case-control clinical trial study has been conducted to comprehensively evaluate the effectiveness of such a treatment modality, partly because pythiosis is a relatively rare disease. Therefore, a multicentric prospective clinical study should be performed to gain insights into the pharmacologic properties of PIA, such as efficacy, adverse effect, optimal dose, and antigen administration. The rabbit model of pythiosis has been established to investigate PIA immunotherapeutic properties [122,250,255,261]. Such an animal model shows atypical manifestations (i.e., cutaneous nodules) compared with pythiosis in the natural hosts (i.e., humans and various animals) [50,97,122,216,224,262,263,264]. Besides, housing the experimental rabbits comes at a high cost, demanding space, facilities, and skilled personnel. These drawbacks impede the use of this model in the evaluation of PIA immunotherapy. On the other hand, the mouse is a well-studied model for the immunological study of many infectious diseases [265,266,267]. Reproduction of the *P. insidiosum* infection in mice is possible but requires pre-treatment with an immunosuppressive agent (i.e., cyclophosphamide) [251,268,269], making this versatile animal model less suitable for the PIA assessment. A murine model of Leishmaniasis (rather than pythiosis) has been developed to evaluate the immunomodulatory properties of PIA, but it does not demonstrate a direct immunological effect of PIA on *P. insidiosum* clearance [256]. Finding a clinically relevant, and cost-effective, animal model would advance our understanding of the properties of the immunotherapeutic PIA.

In the fight against fungal pathogens, several sources of antigens have been under development and assessment, such as live-attenuated or killed organisms, crude fungal extracts, purified or recombinant antigens (i.e., proteins, peptides, carbohydrates, and lipids), and nucleic acids (i.e., DNA and RNA) [242,270]. Regarding the *P. insidiosum* infection, only the crude extract antigens, prepared in several different formulations (i.e., CFA and SABH), are available for immunotherapy of pythiosis. The heat-inactivated organism (i.e., *P. insidiosum* zoospores) has been explored for its in vitro property to stimulate host immunity [252]. A handful of antigens that induce the host immune responses to *P. insidiosum* (so-called immunogens or immunodominant antigens) have been identified by many investigators [37,134,145,271,272,273,274,275,276,277,278,279,280,281]. Reported immunogen profiles are generally inconsistent because different techniques, *P. insidiosum* isolates, and serum samples, were employed (Table 6). Identities, functions, and cellular locations of most immunogens have not been characterized. Recently, Chechi et al. used two-dimensional immunoblotting and mass spectrometry to identify several immunogens (i.e., HSC70, HSP70, exo-1,3-ß-glucanase, fructose-bisphosphate aldolase, and aconitate hydratase) recognized by sera from humans and horses with pythiosis [278]. The *P. insidiosum* genome data [282,283,284,285,286,287] makes it possible to clone the coding sequence for recombinant protein production or synthesize an immunoreactive peptide of the identified immunogens. Several *P. insidiosum* proteins, such as exo-1,3-beta-glucanase (Exo1), elicitin (ELI025), and OPEL-like protein (I06), have been successfully cloned, expressed, and purified from bacterium-based, and cell-free, protein biosynthesis systems [275,277,279,288]. These recombinant proteins could serve as unlimited reproducible antigen sources for the future development of a protein subunit for preventing and treating pythiosis.

Because pythiosis has been increasingly reported worldwide, the disease has become a global healthcare concern. The causative agent, *P. insidiosum*, colonizes ubiquitously on a water plant in the environment [36,37,41]. Once an individual comes in contact with the organism in its habitat, the infection can be initiated, causing a difficult-to-treat disease. The pathological structure called “kunker” formed in *P. insidiosum*-infected animal tissue can give rise to a propagating organism upon exposure to water [42]. Outbreaks of pythiosis have been reported in animals [118,239,240,274] due to the interplay between humans, animals, plants, and their environment. One Health-based approach (as described by the Centers for Disease Control and Prevention; https://www.cdc.gov/onehealth/; accessed on 22 August 2021) should be incorporated into preventive and control measures to promote an optimal health outcome for patients with pythiosis.

Pythiosis is a neglected tropical disease with high morbidity and mortality, in which clinical information on the disease and availability of the preventive, diagnostic, and therapeutic tools are limited. The disease can be considered a part of the Sustainable Developments Goals (SDGs) defined in the United Nations Development Program (https://www.undp.org/sustainable-development-goals/; accessed on 22 August 2021). This review article presents the up-to-date information of the immunotherapeutic PIA for the proper use and future development of this treatment measure, aiming to promote good health and well-being of affected patients. The current formulation of PIA can mitigate morbidity and mortality in humans and animals with pythiosis by reducing surgical intervention and increasing the cure rate. However, more work needs to be done to improve the PIA efficacy in the prevention and treatment of pythiosis.

## 8. Conclusions

The fungus-like organism *P. insidiosum* causes pythiosis, a high morbidity and mortality disease, in humans and animals worldwide. As a part of the treatment, many pythiosis patients received the immunotherapeutic PIA. Only the PIA formulation containing the crude antigenic extracts of *P. insidiosum* has been clinically used over the past decades. Based on 967 documented human and animal pythiosis cases, PIA immunotherapy reduced disease morbidity and mortality. As the final clinical outcomes, 19.4% of PIA-immunized human patients succumbed to vascular pythiosis instead of 41.0% in unimmunized cases. PIA immunotherapy may not provide an advantage in a local *P. insidiosum* infection of the eye. Both PIA-immunized and unimmunized horses with pythiosis showed a similar survival rate of ~70%; however, demands for surgical intervention were much lesser in the immunized cases who were cured (22.8% vs. 75.2%). A case-control clinical trial study should be conducted to evaluate the effectiveness of immunotherapy for pythiosis. The proposed mechanism of the PIA action involves switching the non-protective Th2 to curative Th1 mediated immunity against *P. insidiosum*. Finding a clinically relevant, and cost-effective, animal model of pythiosis is necessary to advance our understanding of the underlying mechanism and the required component of the immunotherapeutic PIA. By exploring the available *P. insidiosum* genome data, synthetic peptides, recombinant proteins, and nucleic acids are potential sources of the immunomodulating antigens worth investigating. The PIA therapeutic property needs improvement for a better prognosis of pythiosis patients.

## Figures and Tables

**Table 1:** Vascular pythiosis patients (147 cases) with or without the *P. insidiosum* antigen (PIA) immunotherapy, reported in the literature during 1989–2021.

Authors	Year of Publication	Country	Study Period	Number of Vascular Cases	PIA-Immunized Cases			PIA-Unimmunized Cases			References
					Number	Survived	Dead	Number	Survived	Dead	
Sathapatayavongs et al. and Tanphaichitra et al.	1989	Thailand	(unknown)	5	-	-	-	5	3	2	[161,162] *
Chetchotisakd et al.	1992	Thailand	1988–1989	4	-	-	-	4	2	2	[163]
Wanachiwanawin et al.	1993	Thailand	1983–1989	6	-	-	-	6	2	4	[110]
Thitithanyanont et al.	1998	Thailand	1995	1	1	1	-	-	-	-	[107]
Prasertwitayakij et al.	2003	Thailand	(unknown)	1	-	-	-	1	1	-	[51]
Wanachiwanawin et al.	2004	Thailand	1998–2002	8	8	6	2	-	-	-	[131]
Pupaibool et al.	2006	Thailand	(unknown)	2	1	-	1	1	1	-	[154]
Laohapensang et al.	2009, 2005	Thailand	2001–2004	7	1	1	-	6	5	1	[50,153] *
Narkwiboonwong et al.	2011	Thailand	(unknown)	1	1	-	1	-	-	-	[108]
Salipante et al.	2012	USA	(unknown)	1	1	1	-	-	-	-	[155]
Keoprasom et al.	2013	Thailand	(unknown)	1	1	1	-	-	-	-	[157]
Schloemer et al.	2013	USA	(unknown)	1	1	-	1	-	-	-	[156]
Hahtapornsawan et al.	2014	Thailand	(unknown)	1	1	1	-	-	-	-	[158]
Pan et al.	2014	USA	(unknown)	1	1	-	1	-	-	-	[7]
Reanpang et al. and Sudjaritruk et al.	2015, 2011	Thailand	2004–2014	22	18	14	4	4	-	4	[149,152] *
Sermsathanasawadi et al.	2016	Thailand	2005–2015	11	10	7	3	1	-	1	[109]
Worasilchai et al.	2018	Thailand	2010–2016	50	50	45	5	-	-	-	[151]
Chitasombat et al. and Khunkhet et al.	2018, 2015	Thailand	2006–2016	13	11	8	3	2	1	1	[2,13,159] *
Susaengrat et al.	2019	Thailand	(unknown)	2	2	2	-	-	-	-	[90]
Torvorapanit et al.	2021	Thailand	2019–2020	9	-	-	-	9	8	1	[164]
Total (%)				147	108 (100%)	87 (80.6%)	21 (19.4%)	39 (100%)	23 (59.0%)	16 (41.0%)	

* These references contain overlapped cases.

**Table 2:** Ocular pythiosis patients (168 cases) with or without the *P. insidiosum* antigen (PIA) immunotherapy, reported in the literature during 1993–2021.

Authors	Year of Publication	Country	Study Period	Number of Cases	PIA-Immunized Cases	Clinical Outcome		References
						Eye Saved	Eye Removed	
Virgile et al.	1993	USA	1989	1	-	1	-	[165]
Murdoch et al.	1997	New Zealand	1984	1	-	-	1	[9]
Badenoch et al.	2001	Australia	(unknown)	1	-	1	-	[166]
Krajaejun et al.	2004	Thailand	1985–2003	11	-	1	10	[167]
Badenoch et al.	2009	Australia	(unknown)	1	-	1	-	[168]
Lekhanont et al.	2009	Thailand	(unknown)	1	1	-	1	[150]
Tanhehco et al.	2011	USA	(unknown)	1	-	-	1	[169]
Barequet et al.	2013	Israel	(unknown)	1	-	1	-	[170]
del Castillo-Jiménez et al.	2013	Spain	2009	1	-	-	1	[171]
Thanathanee et al.	2013	Thailand	2009	3	3	2	1	[132]
Hung et al.	2014	Canada	(unknown)	1	-	-	1	[8]
Sharma et al.	2015	India	2010–2012, 2014	11	-	9	2	[172]
Lelievre et al.	2015	France	(unknown)	1	-	1	-	[173]
Ramappa et al.	2016	India	2016	1	-	1	-	[97]
He et al.	2016	China	(unknown)	1	-	1	-	[54]
Ros Castellar et al.	2017	Spain	(unknown)	1	-	1	-	[174]
Raghavan et al.	2018	India	(unknown)	1	-	1	-	[175]
Agarwal et al.	2018	India	2014–2016	10	-	8	2	[176]
Chatterjee et al.	2018	India	2017	1	-	1	-	[96]
Anutarapongpan et al.	2018	Thailand	2009–2015	21	-	6	15	[177]
Neufeld et al.	2018	USA	(unknown)	1	-	-	1	[178]
Rathi et al.	2018	India	(unknown)	1	1	-	1 *	[1]
Maeno et al.	2019	Japan	(unknown)	1	-	1	-	[53]
Bernheim et al.	2019	France	(unknown)	1	-	1	-	[47]
Permpalung et al.	2019	Thailand	2010–2016	30	30	16	14	[127]
Wang et al.	2020	China	2008–2017	4	-	1	3	[179]
Puangsricharern et al.	2021	Thailand	2006–2019	9	-	3	6	[180]
Gurnani et al.	2021	India	2017–2020	26	-	26	-	[181]
Vishwakarma et al.	2021	India	2016–2018	18	-	15	3	[182]
Nonpassopon et al.	2021	Thailand	2009–2019	6	-	5	1	[183]
Total (%)				168 (100%)	35 (20.8%)	104 (61.9%)	64 (38.1%)	

* Deceased.

**Table 3:** Clinical details and treatment outcomes of vascular pythiosis patients with (67 cases) or without (38 cases) the *P. insidiosum* antigen (PIA) immunotherapy.

Clinical Features Presented as % [Case Ratio]	PIA-Immunized Cases (*n* = 67)	PIA-Unimmunized Cases (*n* = 38)
1. Age (years)	40.9 (range: 10–75)	41.4 (range: 17–73)
2. Male:Female	5:1	2.4:1
3. Underlying hematologic disorder	91.0 [61/67]	100.0 [38/38]
4. Infection site		
Head and neck	6.0 [4/67]	-
Upper limb	1.5 [1/67]	-
Lower limb	92.5 [62/67]	100.0 [38/38]
Aorta/Iliac artery	33.9 [21/62]	44.7 [17/38]
Femoral/Popliteal artery	53.2 [33/62]	52.6 [20/38]
Tibial/Peroneal/Dorsalis pedis	12.9 [8/62]	2.6 [1/38]
5. Limb amputation		
Survived	61.2 [41/67]	57.9 [22/38]
Dead	23.9 [16/67]	31.6 [12/38]
6. No surgical amputation		
Survived	9.0 [6/67]	0.0 [0/38]
Dead	6.0 [4/67]	10.5 [4/38]
7. Mortality rate based on infection site		
Overall	29.9 [20/67]	42.1 [16/38]
Head and neck	50.0 [2/4]	-
Upper limb	0.0 [0/1]	-
Lower limb	29.0 [18/62]	42.1 [16/38]
Aorta/Iliac artery	61.9 [13/21]	88.2 [15/17]
Femoral/Popliteal artery	15.2 [5/33]	5.0 [1/20]
Tibial/Peroneal/Dorsalis pedis	0.0 [0/8]	0.0 [0/1]
8. Disease duration before treatment	(*n* = 51) *	(*n* = 34) *
≤2 months	28.6 [6/21]	25.0 [5/20]
>2 months	33.3 [10/30]	50.0 [7/14]

* The number of cases whose data about disease duration before initiation of the treatment are available.

**Table 4:** Animals with pythiosis (270 cases) receiving the *P. insidiosum* antigen (PIA) immunotherapy during 1981–2021.

Authors	Year of Publication	Country	Study Period	Affected Animal	Infected Tissue	PIA-Immunized Cases	Clinical Outcome		References
							Cured	Unresponsive or Dead	
Miller RI.	1981	Australia	1977–1981	Horse	Skin	40	33	7	[119]
Miller et al.	1983	USA	(unknown)	Horse	Skin	5	1	4	[126]
Miller et al.	1985	USA	1983	Calf	Skin	1	-	1	[133]
Mendoza et al.	1992	Costa Rica	1982–1988	Horse	Skin	71	47	24	[123]
Duncan et al.	1992	USA	(unknown)	Cat	Skin	1	-	1	[27]
Eaton SA.	1993	USA	(unknown)	Horse	Skin, Bone	1	-	1	[198]
Thomas et al.	1998	USA	(unknown)	Cat	Skin	1	-	1	[194]
Dowling et al.	1999	Australia	(unknown)	Horse	Skin	2	1	1	[199]
Dykstra et al.	1999	USA	1985–1995	Dog	Skin	2	-	2	[191]
Santurio et al.	2001	Brazil	1996–1997	Horse	Skin	35	26	9	[144]
Reis et al.	2003	Brazil	1996–1999	Horse	Skin, Lung, Liver	1	-	1	[58]
Hensel et al.	2003	USA	(unknown)	Dog	Skin	1	1	-	[192]
Mendoza et al.	2003	USA	(unknown)	Horse	Skin	18	13	5	[121]
				Dog	Skin	5	2	3	
				Dog	Intestine	1	-	1	
Wellehan et al.	2004	USA	(unknown)	Camel	Skin	1	-	1	[29]
Mendoza et al.	2004	USA	(unknown)	Cat	Skin	1	-	1	[137]
White et al.	2008	USA	(unknown)	Horse	Skin	1	-	1	[139]
de Faria Maciel et al.	2008	Brazil	2006–2007	Horse	Skin	1	-	1	[200]
Frey Jr. et al.	2007	Brazil	2004–2005	Horse	Skin, Bone	10	5	5	[201]
Bandeira et al.	2009	Brazil	(unknown)	Horse	Skin	1	-	1	[202]
dos Santos et al.	2011	Brazil	2007–2009	Donkey	Skin	1	-	1	[128]
de Avila et al.	2011	Brazil	2010	Horse	Skin	1	1	-	[197]
Schmiedt et al.	2012	USA	(unknown)	Dog	Intestine	1	1	-	[140]
Videla et al.	2012	USA	2009	Camel	Skin	2	1	1	[124]
Pereira et al.	2013	Brazil	(unknown)	Dog	Intestine	1	1	-	[129]
Carrera et al.	2013	Brazil	2011	Sheep	Nasal	6	1	5	[193]
dos Santos et al.	2014	Brazil	2007–2010	Horse	Skin	47	38	9	[46]
Oldenhoff et al.	2014	USA	2012	Dog	Skin	1	-	1	[141]
Grant et al.	2016	USA	(unknown)	Calf	Skin	1	1	-	[130]
Gaddis et al.	2017	USA	(unknown)	Dog	Skin	1	-	1	[125]
Silva et al.	2018	Brazil	(unknown)	Horse	Skin	1	-	1	[203]
Dowst et al.	2019	USA	(unknown)	Cat	Skin	1	-	1	[25]
Parambeth et al.	2019	USA	(unknown)	Dog	Intestine	1	-	1	[57]
Di Filippo et al.	2020	Brazil	(unknown)	Horse	Skin, Bone	1	-	1	[204]
Cridge, et al.	2020	USA	2018–2019	Dog	Intestine	1	1	-	[205]
da Paz et al.	2021	Brazil	2018	Horse	Skin	3	2	1	[118]
Total (%)						270 (100%)	176 (65.2%)	94 (34.8%)	

**Table 5:** Treatment outcomes of pythiosis in animals with (270 cases) or without (377 cases) the *P. insidiosum* antigen (PIA) immunotherapy.

Animal Species	PIA-Immunized Cases			PIA-Unimmunized Cases			
	Number of Cases	Cured	Unresponsive or Dead	Number of Cases	Cured	Unresponsive or Dead	References
Horses	239 (100%)	167 (69.9%)	72 (30.1%)	170 (100%)	125 (73.5%)	45 (26.5%)	[19,46,49,58,87,89,92,93,94,105,118,207,208,209,210,211,212,213,214]
Dogs	15 (100%)	6 (40.0%)	9 (60.0%)	75 (100%)	17 (22.7%)	58 (77.3%)	[6,20,21,23,45,55,56,85,141,191,205,215,216,217,218,219,220,221,222,223,224,225,226,227,228,229,230,231,232,233,234,235]
Cows	2 (100%)	1 (50.0%)	1 (50.0%)	128 (100%)	127 (99.2%)	1 (0.8%)	[133,236,237,238,239,240,241]
Sheep	6 (100%)	1 (16.7%)	5 (83.3%)	1 (100%)	-	1 (100.0%)	[193]
Cats	4 (100%)	-	4 (100.0%)	2 (100%)	1 (50.0%)	1 (50.0%)	[48,194]
Donkeys	1 (100%)	-	1 (100.0%)	1 (100%)	1 (100.0%)	-	[44]
Camels	3 (100%)	1 (33.3%)	2 (66.7%)	-	-	-	[29,124]
Total	270 (100%)	176 (65.2%)	94 (34.8%)	377 (100%)	271 (71.9%)	106 (28.1%)	

**Table 6:** Immunodominant antigens identified in *P. insidiosum* by Western blot analysis.

Authors	Year of Report	Type of Antigen *	Source of Antigen or Isolate (Number, Country) **	Source of Pythiosis Sera (Number; Country) ***	The Molecular Weight of Immunogen (kDa)	References
Mendoza et al.	1992	SABH	Horses (4, Australia, Costa Rica, Japan, USA); Human (1, Thailand)	Horses (5, Costa Rica)	28, 30, 32	[134]
Vanittanakom et al.	2004	CFA	Human (1, Thailand)	Human (1, Thailand); Rabbit (1, unknown)	23, 26, 28–30, 42–35, 56, 73, 110	[276]
Leal et al.	2005	SABH	Horse (1, Brazil)	Horses (3, Brazil); Cows (2, Brazil); Rabbits (2, unknown)	33.5, 35, 38, 39, 40, 70, 80	[273]
Perez et al.	2005	SABH	Horse (1, Costa Rica)	Calves (57, Venezuela)	30–32, 51–203	[274]
Krajaejun et al.	2006	CFA, SABH	Humans (16, Thailand)	Humans (12, Thailand)	34–43, 74	[271]
Supabandhu et al.	2008	CFA	Environment (7, Thailand)	Rabbit (1, unknown)	35–40, 70	[37]
Chindamporn et al.	2009	SABH	Horses (4, New Guinea, Australia, Costa Rica, USA); Humans (2, Thailand)	Human (1, USA); Humans (2, Thailand); Horses (2, USA); Horse (1, Costa Rica); Dogs (3, USA); Cows (3, Venezuela); Cats (3, USA)	23, 28, 30, 32, 34, 41, 46, 49, 51, 60, 74, 76, 78, 80, 124, 209	[145]
Lerksuthirat et al.	2015	Recombinant ELI025 protein	*E. coli*-based protein synthesis	Humans (3, Thailand); Rabbit (1, unknown)	12.4	[279]
Keeratijarut et al.	2013, 2015	Exo1 peptides	Peptide synthesis (Peptide-A and -B)	Humans (34, Thailand)	(14, 15 amino acids)	[280,281]
Dal Ben et al.	2018	SABH	Horses (20, Brazil); Dogs (2, Brazil)	Horse (1, Brazil); Dog (1, Brazil); Cow (1, Brazil); Rabbit (1, unknown)	24, 34, 50–55, 60	[272]
Sae-Chew et al.	2020	Synthesized I06 protein	Cell-free protein synthesis	Humans (21, Thailand)	55	[275]
Rotchanapreeda et al.	2020	Recombinant Exo1 protein	*E. coli*-based protein synthesis	Humans (12, Thailand)	82	[277]
Chechi et al.	2021	SABH	Horse (1, Brazil)	Horses (22, Brazil); Humans (10, Thailand)	25, 28, 31, 35, 37, 38, 43, 48, 53, 57, 63, 68, 69, 86, 88	[278]

* CFA (culture filtrate antigens) represents extracellular proteins; SABH (soluble antigens from broken hyphae) represents intracellular proteins. ** Number and geographic distribution (countries) of *P. insidiosum* isolated from humans and animals with pythiosis and used to prepare CFA and SABH for Western blot and ELISA analyses. *** Number and geographic distribution (countries) of pythiosis sera, derived from humans and animals with pythiosis, and used for Western blot and ELISA analyses.

## Data Availability

Not applicable.
